# A review of self-healing mechanisms for the application of conductive electronic products

**DOI:** 10.1039/d5ra08645k

**Published:** 2026-02-18

**Authors:** Wei Wuen Ng, Wei-Hsin Chen, Hui San Thiam, Steven Lim, Yi-Kai Chih, Yean Ling Pang

**Affiliations:** a Lee Kong Chian Faculty of Engineering & Science, Universiti Tunku Abdul Rahman, Sungai Long Campus Jalan Sungai Long, Bandar Sungai Long Selangor 43000 Malaysia; b Department of Aeronautics and Astronautics, National Cheng Kung University Tainan 701 Taiwan weihsinchen@gmail.com chenwh@mail.ncku.edu.tw; c Department of Chemical and Materials Engineering, Tunghai University Taichung 407 Taiwan; d Department of Mechanical Engineering, National Chin-Yi University of Technology Taichung 411 Taiwan; e Centre for Advanced and Sustainable Materials Research, Universiti Tunku Abdul Rahman, Sungai Long Campus Jalan Sungai Long, Bandar Sungai Long Selangor 43000 Malaysia; f Department of Chemical and Materials Engineering, National University of Kaohsiung Kaohsiung 811 Taiwan

## Abstract

The rapid growth of electronic devices has raised concerns about durability and e-waste. Self-healing materials offer a promising solution by extending device lifespan and reducing maintenance needs. This review examines the use of self-healing materials in conductive electronic systems, focusing on intrinsic and extrinsic mechanisms. Extrinsic methods use microcapsules or vascular systems to release healing agents, while intrinsic approaches rely on reversible bonds, such as hydrogen bonding, ionic interactions, and metal coordination, to enable repeatable repair. Applications discussed include strain sensors, energy storage, and flexible electronics. The review highlights how these materials improve reliability in wearable devices, energy harvesters, and wireless systems. Critical factors affecting healing performance, like healing time, environment, and material design, are also analyzed. This review serves as a useful reference for selecting suitable self-healing strategies for next-generation electronic applications.

## Abstract 2

This review introduces intrinsic and extrinsic self-healing electronic materials. Dynamic bonds and healing agents restore function after damage, enabling durable flexible sensors, wearable electronics, and energy devices.

## Graphical abstract

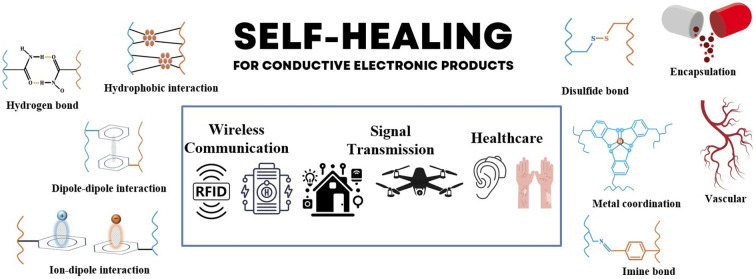

## 1. Introduction

**Fig. 1:**
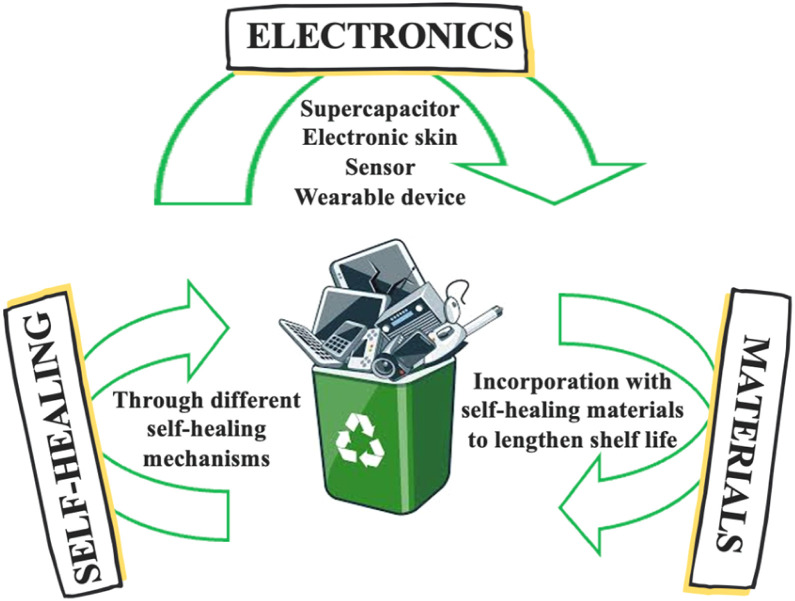
Advances in electronic products.

In today's rapidly transforming digital landscape, the rapid industrialization and globalization of the modern world have accelerated the demand for electronic devices. Electronic devices extend beyond our daily lives, from sensors in healthcare to supercapacitors and energy generators, implicitly suggesting to mainstream society that they have become our inseparable companions. Nevertheless, mechanical damage (scratches, drops, impacts) and operational fatigue have been persistent challenges, as they lead to rapid obsolescence and shorter lifespans of electronic devices.^1,2^ The inability of products to maintain their initial functionality results in significant electronic waste (e-waste). In this context, the UN's Fourth Global E-waste Monitor (GEM) found that global e-waste generation is growing five times faster than recorded e-waste recycling.^3^ Uncontrolled e-waste has induced detrimental environmental pollution and human health impacts. Thus, as sustainability and environmental concerns take centre stage, scientists and engineers are striving to develop innovative solutions to achieve an exceptional future of longevity and resilience. Cutting-edge concepts, such as self-healing ability (Fig. 1), represent a groundbreaking advancement and hold immense potential to revolutionize various sectors, particularly by addressing the limited service life commonly associated with wearable devices.^4^

The development of self-healing materials has progressed rapidly and they have found diverse applications due to their innovative nature and distinct advantages over conventional materials. These materials have the potential to repair or mend themselves, capable of recovering their functionality upon cracks or damage.^5^ Self-healing materials thereby not only retain their original functions, thereby increasing the product's service period, but also save costs due to reduced maintenance and higher safety standards. This technology has proven to be a successful strategy for reducing waste. Acknowledging this, the self-healing technology has been applied across numerous industries, including automotive, aerospace, construction, and electronics.^6,7^

The concept of self-healing primarily originates from regenerative behaviour in biological systems capable of autonomous damage repair. Self-healing materials were first translated into synthetic materials in the late 20th/21st Century, between the 1980s and 1990.^8,9^ During this period, the research was largely concentrated on polymers and composites. A major milestone was achieved in 2001 with the demonstration of self-healing polymers following the development of the microcapsule-based system,^10^ which established a practical foundation for intrinsic and extrinsic healing mechanisms. Building on these advances, the 2010s saw rapid integration of self-healing materials into electronic systems, particularly flexible and stretchable electronics, where mechanical reliability is critical.^11–13^ More recently, research in the 2020s has focused on advanced applications across diverse electronic fields, alongside the development of hybrid self-healing strategies that combine multiple mechanisms to achieve enhanced durability and multifunctionality.^14^ A unique architecture was reported by Liu *et al.*,^14^ which combined extrinsic and intrinsic self-healing mechanisms to synergistically improve healing efficiency and mechanical properties. On the other hand, Wen *et al.*^15^ made use of two different interactions, including dynamic disulfide and hydrogen bonds, for self-healable flexible sensors for monitoring human motion.

As robotic engineering systems become prevalent in the era of Industry 4.0, the technological revolution is driving greater flexibility and efficiency in machines and electronics.^16^ However, the lack of resilience in traditional electronic materials accelerates product failure, leading to frequent replacements and contributing to environmental pollution. Given the pressing need to enhance device longevity while reducing waste, self-healing materials offer a promising avenue for an innovative solution. Self-healing materials resemble living organisms, naturally repairing damage and restoring functionality. A lot of research has been devoted to investigating the potential of self-healing materials in electronic products, such as smart robotics, wearable electronics, and health monitoring systems, with the aim of enabling self-healing behaviour and reinforcing material durability.^17^ One of the key aspects of this innovation lies in its potential to drive economic growth, enhance productivity, and foster technological diffusion. Although significant progress has been made in developing self-healing conductive electronic products, comprehensive reviews discussing these breakthroughs from a fundamental perspective are still lacking. This paper aims to fill that gap by providing a consolidated overview of self-healing strategies in electronics, with a focus on approaches that enable damage repair in ambient conditions and extend the operational lifespan of electronic products.

## 2. Chemistry, strategies and mechanism of self-healing

Through intelligent chemical architecture, a self-healing conductive material can detect damage, self-heal, and recover its original mechanical, electrical, or operational function. To endow these properties, polymers and composites should:

(a) *Incorporate dynamic reversible interactions*

Interactions include ionic interactions, hydrogen bonding, metal–ligand coordination, or dynamic covalent bonds, enabling autonomous bond dissociation and reformation after damage.

(b) *Polymer chain mobility*

Chain mobility is essential to facilitate interdiffusion across damaged interfaces. Sufficient segmental mobility enables functional groups to re-engage and reconstruct the material network; however, excessive mobility may compromise mechanical strength and dimensional stability. Therefore, optimizing crosslink density and interaction strength is critical.

(c) *Reformation of conductive pathways*

In conductive systems, an additional challenge is reconstructing conductive pathways without compromising mechanical robustness. This often leads to inherent trade-offs between conductivity, mechanical strength, and healing efficiency, necessitating careful material and structural design. Addressing these challenges remains a key focus for advancing next-generation self-healing electronic devices.

The design of self-healing conductive systems requires a careful balance between healing capability and functional performance.^18^ Healing must effectively restore conductivity without compromising key electrical or mechanical properties and should occur under practical operating conditions without requiring complex external intervention.^19^

**Fig. 2:**
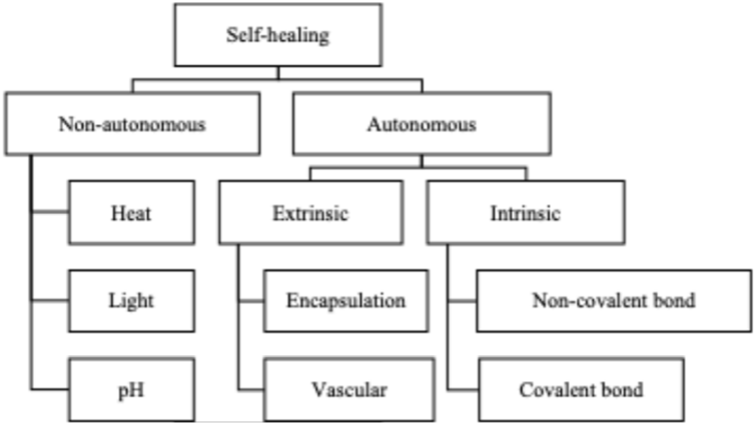
Classification of self-healing mechanisms.

Generally, self-healing materials can be expeditiously categorized into two groups (as shown in Fig. 2): non-autonomous healing, which requires an external stimulus, and autonomous healing, which occurs spontaneously.^20^ The so-called non-autonomous self-healing materials are stimuli-responsive, in which self-healing is induced by external light or heat. In contrast, autonomous healing systems require no stimulus, and self-healing occurs promptly when damage occurs.^21^ Based on the presence of healing agents, self-healing materials can be further classified into extrinsic and intrinsic types. The extrinsic self-healing system involves storing self-healing agents in carriers (microcapsules or microvessels), and delivering them to damaged locations as soon as damage is inflicted.^22^ Intrinsic self-healing materials possess an inherent ability to heal themselves through reversible chemical bonds or physical interactions.

While most reviews focus on non-autonomous self-healing systems, only a few attempts have been made to summarize the functionalities of autonomous self-healing systems.^23–25^ It is crucial to delve into the role of self-healing materials in the electronic world and understand the actual value these devices have contributed since their inception. Electronic devices can generally be divided into three categories: insulators, semiconductors, and conductors, with conductors further classified into electronic and ionic conductors.^26,27^ Accordingly, this review discusses the self-healing mechanisms exhibited by different material classes, their potential applications in various electronic-conductive scenarios, and highlights self-healing efficiency across key characteristics, including stretchability, mechanical toughness, and electrical conductivity.

### 2.1. Extrinsic self-healing

Extrinsic self-healing is exemplified by capsule- or vessel-based internal carriers that enable self-healing without altering the matrix's structure. Healing agents, usually reactive chemical reagents (precursors or catalysts), are embedded in the materials as microcapsules.^28^ When damage occurs and cracks propagate, the protective shell of the capsule ruptures, followed by the release and delivery of repair chemicals to the damaged areas, allowing the void spaces to be filled *via* capillary action.^29^ The released chemicals initiate chemical reactions to regenerate the bond, thereby joining the rupture parts. Vascular-based self-healing uses a vascular kind of storage for self-healing agents and operates similarly to capsular-type self-healing. Capsule-based self-healing is applied to microscopic damage since the capsule stores only a small amount of healing agent, while vascular-based self-healing can heal larger damage and reach distant damage points.^22,30^ The healing efficiency of both systems is affected by dynamic factors, such as pressure and flow rate, that influence the rupture and mixing of the healing agent with the damaged material. The healing capacity also depends on the amount of healing agents, as these resources are depleted during the repair process, restricting secondary healing and ultimately diminishing healing ability.

#### 2.1.1. Encapsulation

In encapsulation, extrinsic self-healing, microcapsules filled with healing agents are embedded in the electronic products. The microcapsules protect the healing agent from premature release and remain functional until they are triggered by damage. When mechanical damage occurs, stress or bending causes the capsule to rupture, releasing the healing agent into the damaged area. The healing agent flows to the crack spots and reseals the damaged material, allowing the electronic devices to continue functioning without external repairs.

Allahdini *et al.*^31^ reported a self-healing composite as an anti-icing coating for electrical products, using polydimethylsiloxane (PDMS) as the healing agent and dibutyltin dilaurate (DBTL) as the catalyst. When the mechanical damages propagated until where the PDMS capsule was located, the cracks triggered the capsule to rupture. The PDMS was directed to the damaged area to initiate the polycondensation reaction. The PDMS dispersed throughout the matrix, forming a highly crosslinked polymeric network. The self-healing efficiency was evaluated by tensile testing, and the material recovered approximately 67% of its initial tensile strength.

The movement of lithium ions through the battery electrodes causes fractures and debonding, disrupting the battery's conductivity. Thus, Odom *et al.*^32^ proposed a self-healing anode by embedding a microcapsule consisting of conductive carbon nanotubes to restore electrical performance after mechanical damage. The microcapsule size was maintained between 125 to 180 µm. The circuit was subjected to controlled, repeatable failure by applying a load with a 4-point bend setup and allowing it to heal for 5 minutes. Full restoration was observed through the conductivity analysis, demonstrating effective self-healing of carbon nanotubes.

Blaiszik *et al.*^33^ introduced a healing material composed of gallium–indium (Ga–In) liquid metal in a dielectric material. The crack was initiated at a critical bending load, triggering the healing capsule to rupture and release. The healing agent was then liberated to the cracked area once the circuit was broken. The circuit was almost recovered, achieving 99% healing efficiency in terms of voltage within 1 ms.

Microcapsule-based systems are excellent for targeted, one-time damage repair, especially in applications like circuit healing, where speed and precision are critical. However, their single-use nature limits long-term durability unless reloaded or supported by redundant capsules.

#### 2.1.2. Vascular

Similar to blood vessels, vascular materials form a three-dimensional (3D) structure with interconnected channels, allowing rapid diffusion of self-healing agents to the damaged areas. A 3D branching network is formed within the material using a sacrificial template, which is later removed, leaving hollow channels.^34^ The channels are then filled with healing agents through inlet ports. The 3D interconnected network of microchannels allows healing agents to flow throughout the material *via* pumps or capillary action, reaching deep or internal cracks.

Bekas *et al.*^35^ prepared self-healing vascular fiber-reinforced polymer (FRP) composites by incorporating multiwall carbon nanotubes (MWCNTs) as the healing agent within the polymeric matrix. The vascular network was created by embedding nylon strings at the mid-plane of the laminate, aligned parallel to the fiber direction. After curing, the nylon strings were manually removed, leaving continuous hollow channels within the composite that served as the vascular network. Healing agents were injected into the fractured region through the vascular channels using a syringe pump. Experiment results showed that FRP doped with 0.3 wt% of MWCNT increased the fracture toughness of pristine FRP by 33.8%. Interestingly, the SEM image showed that the composite could close the fracture, indicating that MWCNT conferred self-healing by providing adhesive strength between the FRP and MWCNT. In the study, the composite fully recovered mechanical fracture toughness, demonstrating 169% self-healing efficiency, because the MWCNTs developed a stronger interfacial region through interaction with the FRP, improving stress distribution and enhancing fracture toughness during healing.

Hart *et al.*^36^ studied the healing of delamination damage in an epoxy composite by polymerizing amine-based reactants within the epoxy system. Fracture testing was performed to study the healing efficiency of the epoxy/amine composite. The composite underwent multiple healing cycles, and it restored 95% of its original fracture toughness at the first cycle. While increasing the healing cycles to 10 times, the healing efficiency dropped to 67% because the healing agents (resin and hardener) were not well mixed, resulting in poor distribution and insufficient contact, thereby reducing polymerization effectiveness. Additionally, residual damage on the fracture surfaces contributes to the decline in performance over time.

While encapsulated healing agents offer a clever and compact solution, the long-term stability and capacity for repeated self-healing remain questionable. In contrast, vascular systems can enable larger-area damage repair, but their complexity and fabrication challenges limit their integration into compact and miniaturized devices.

### 2.2. Intrinsic self-healing

**Fig. 3:**
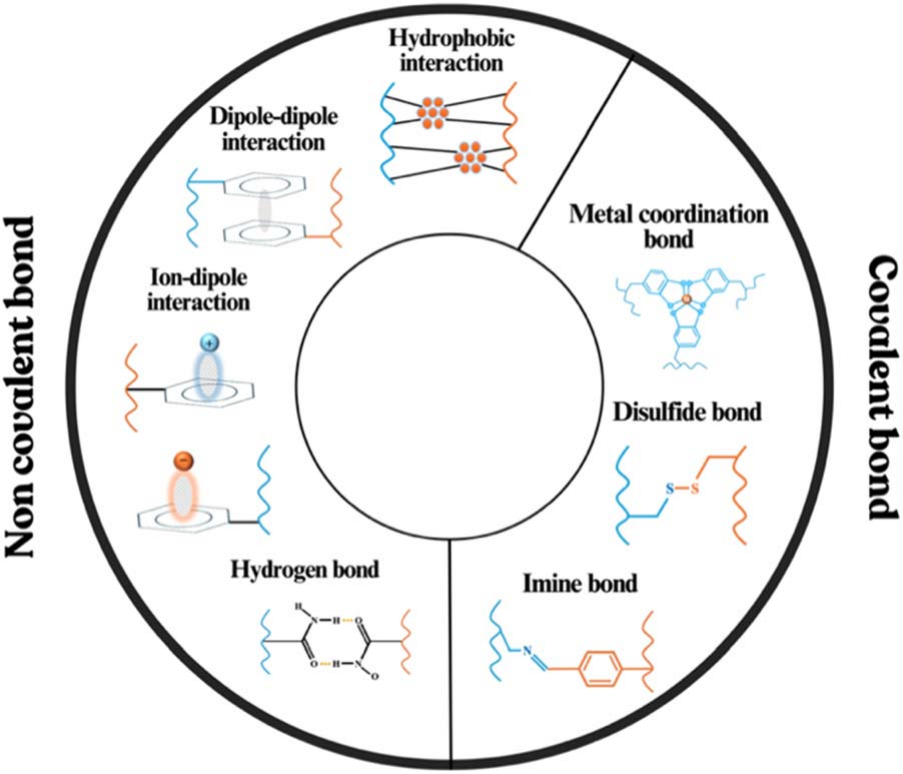
Covalent and non-covalent interactions enabling self-healing in electronic devices.

Intrinsic self-healing materials employ the inherent specific and reversible interactions (Fig. 3) between the molecular constituents within the structure.^37^ Intrinsic healing overcomes the problem of limited repair cycles in extrinsic systems through reversible bonding, enabling continuous detection and damage repair. Thus, the intrinsic self-healing approach allows materials to recover their properties multiple times after damage, marking a promising trial in self-healing electronics.

#### 2.2.1. Non-covalent interaction

The self-healing behaviour through non-covalent bonding relies on weak, reversible interactions. These weak reversible interactions will break under uncontrolled stress, generating newly free groups (functional groups on the polymer chains that were previously involved in non-covalent interactions). The dynamic bonds will be rebuilt and reconnected automatically in unstressed conditions at ambient temperature, driven by the reversible reaction, without external stimulus.^38^ Hydrogen bonds, ionic interactions, and metal-coordination bonds are the most commonly adopted non-covalent interactions. For example, when mechanical stress includes fracture, the hydrogen bonds break, but the polar groups remain active. Upon realignment of the chains in unstressed conditions, hydrogen bonds readily reform, enabling the material to recover its structure.

##### 2.2.1.1. Hydrogen bonding

Hydrogen bonding (<40 kJ mol^−1^)^39^ is deemed the most ubiquitous supramolecular interaction that confers self-healing functionality owing to the abundant choice of materials.^40–42^ Hydrogen bonds are formed between a partially positive hydrogen atom and an unpaired, highly electronegative atom (F, O, or N).^43^ When the hydrogen bond breaks, the broken hydrogen bonds are promptly attracted to another missing part at the cut interfaces, leading to bond restoration and rapid, highly efficient self-healing.

**Fig. 4:**
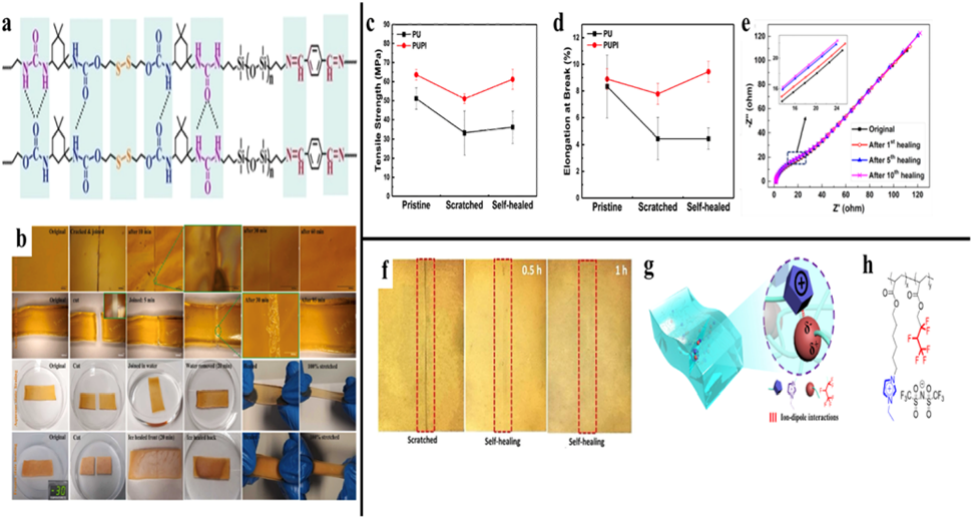
(a) Hydrogen-bonded network of the self-healed T-hydrogel-based strain sensor, reproduced with permission from *Chemistry – A European Journal*,^44^ copyright 2023. (b) Optical image of a hydrogel being healed in different conditions, reproduced with permission from *Nano Energy*,^46^ copyright 2023. (c and d) Tensile strength and elongation at break of PU and PUPI before and after self-healing, reproduced with permission from *Macromolecules*,^1^ copyright 2020. (e) Ionic conductivity of the hydrogel after multiple cycles of self-healing, reproduced with permission from *Chemistry of Materials*,^50^ copyright 2018. (f) Image of the damaged and healed conductive hydrogel, reproduced with permission from *Chemical Engineering Journal*,^53^ copyright 2024. (g and h) Structure and design of intrinsically conductive hydrogel, reproduced with permission from *ACS Applied Materials & Interfaces*,^54^ copyright 2021.

Most electronic gadgets include strain sensors as essential components, as they convert strain and stress information into electrical impulses. Strain sensors need to be flexible and stretchable to enable better interaction between people and machines, such as controlling devices with gestures. Generally, flexible substrates and conductive materials are the key components of flexible strain sensors. Flexible substrates are base materials that provide support, such as rubber, polydimethylsiloxane (PDMS), polyurethane (PU), fabrics, and thermoplastic elastomer, while conductive materials, which allow electricity to flow through the sensor, including carbon nanotubes, graphene, MXene, and conducting polymer.^44^ However, most stretchable electronic devices will fail after physical damage. To improve device performance, Zheng *et al.*^45^ created a stable, sensitive, and conductive hydrogel (T-hydrogel) using polyvinyl alcohol (PVA) and Ti_3_C_2_T*x* MXene nanosheets, forming a self-healable strain sensor. The resulting T-hydrogel exhibited enhanced electrical conductivity. Integration of MXene in PVA contributed to a compacted hydrogen-bonded network (Fig. 4(a)), yielding a sensor with high mechanical stability and self-healing character in the ambient environment. The cut hydrogel recovered autonomously within 1 hour at ambient conditions and regained its stretchability. The self-healing T-hydrogel-based strain sensor demonstrated desirable self-healing performance, achieving 96.7% recovery in conductivity. This self-healing behavior is attributed to the mobility of the broken PVA chains and the dynamic reformation of hydrogen bonding. Notably, the device demonstrates resilience in detecting bodily motion, potentially offering an innovative approach to human health monitoring and human–computer interaction.

Building on advances in self-healable materials for stretchable electronics, similar strategies are being explored in other wearable systems, such as triboelectric nanogenerators (TENGs), which also rely on flexible and durable materials to maintain performance across various conditions. The induction layer, which is usually a hydrogel, in TENGs collects the charge, transfers it *via* bonding or dipolar interactions, and induces charges in the conductive area. However, it might not always work well due to its high water dependence, which causes it to lose water through evaporation, mechanical deformation, and insufficient protection against moisture loss. This water loss leads to decreased flexibility, lower conductivity, and a less effective induction layer over time. Also, these devices face daunting challenges, including liquid puncturing, interfacial etching, and short-circuiting. Thus, Firdous *et al.*^46^ grafted acrylic acid (AA) onto gum arabic (GA) to develop a stretchable, transparent, self-healable solid hydrogel network for TENG as a power backup in freezing environments. The hydrogel demonstrated remarkable self-healing across various physical states, including dry, aqueous, and frozen conditions (Fig. 4(b)). The hydrogel functioned as a triboelectric nanogenerator when in contact with skin, achieving a power density of 11.1 W m^−2^ at a low matching impedance of 9 MΩ. This performance was maintained even in aqueous (7.28 W m^−2^) and freezing states (7.04 W m^−2^), showcasing its potential for wearable electronic applications. Pandey *et al.*^47^ created a self-healing TENG for self-powered wearable electronics, such as a sensor for monitoring human joint movement. The TENG was fabricated through the hybrid polymerization of Ecoflex and polyborosiloxane (PBS), where the Ecoflex provides elasticity and mechanical robustness, while hydrogen bonding in the PBS enable self-healing capability. The Ecoflex exhibits high tensile strength, outstanding stretchability (590%), and autonomous mechanical self-healing efficiency of 68% within 2 h.

A common method for creating stretchable electrodes is screen printing, in which conductive materials are applied to the surface of the elastomer. However, there are still challenges with the printing method, including the conductive ink not sticking well and the printed layer losing its ability to conduct electricity when overstretched. Upon understanding these problems, Zhan *et al.*^48^ demonstrated a self-healing stretchable electronic device, which is crucial for bio-signal monitoring and intelligent packaging applications. This innovation utilized a combination of liquid metal/silver fractal dendrites and polystyrene-*block*-polyisoprene-*block*-polystyrene (LM/Ag FDs/SIS) conductive inks to improve printability. These materials were screen-printed onto a self-healing thermoplastic polyurethane (TPU) film, forming a self-healing stretchable electrode. The material exhibited high electrical conductivity of 1.6 × 10^6^ S m^−1^. This level of conductivity is essential for effective bio-signal monitoring, ensuring that the devices can accurately transmit signals.

Among self-healing materials, polyurethane (PU) can only repair minor damage, such as dents, but struggles with more significant damage, such as cracks and tears. This limitation often compromises its mechanical properties, which undermines its practical applications in flexible display technology. With this in mind, Lee *et al.*^1^ showed a favourably self-healing system by combining functional polyimide (PI) with conventional polyurethane (PU). The polymeric system demonstrated improved mechanical properties, with the PI chain serving as a polymeric adhesive within the PU matrix, resulting in a remarkable enhancement of intermolecular interactions between PI and PU. That being so, the compromise between the mechanical properties of self-healing materials and their self-healing capability could be realised. By examining the post-self-healing mechanical properties, the tensile strength and elongation at break (Fig. 4(c) and (d)) of the as-fabricated sample approached those of the pristine condition. The use of polymeric glue displayed a prospective strategy for engineering next-generation functional self-healing materials. In addition, these materials demonstrated exceptional self-healing capabilities alongside enhanced mechanical properties, making them excellent candidates for flexible displays and electronic devices.

Song *et al.*^49^ report a thermoelectric polymer composite (TPC) inspired by the skin's thermal sensory system, which simultaneously exhibits thermoelectric, self-healing, and stretchable properties. The TPC, composed of a self-healing polymer matrix, conductive nanofillers, and inorganic thermoelectric particles, sustains extreme mechanical deformation up to 1197% strain and demonstrates efficient self-healing behavior. The composite generates an electrical voltage in response to temperature changes, with its conductivity, Seebeck coefficient, and power factor recovering to over 90% after damage. Furthermore, the temperature-dependent voltage output was successfully used to control a robotic hand, demonstrating a modular, self-bonding e-skin platform with potential for advanced thermal sensing and human–machine interaction.

##### 2.2.1.2. Ionic interaction

Ionic interactions result from electrostatic attraction between ions of opposite charge. The energy associated with ionic interactions exceeds that of hydrogen bonding, making ionic interactions relatively stronger. However, despite their strength, the weak ionic bonds are also reversible and dynamic, which plays a significant role in self-healing. Charged polymer chains exhibit ionic interaction, thus rendering electrical conductivity. Wang *et al.*^50^ employed γ-radiation to incorporate graphene oxide into soluble starch and poly(sodium 4-vinyl-benzenesulfonate-*co-N*-(2-(methacryloyloxy)ethyl)-*N*,*N*-dimethylbutan-1-aminium bromide), forming a self-healable double-network hydrogel. The hydrogel exhibited high conductivity and strong adhesion. The self-healing test showed that the separated hydrogel pieces could attach to one another within 1 day at ambient temperature. After that, rheology analysis was executed to quantify the self-healing activity. The rheological results revealed that the storage modulus of the repaired gel rapidly returned to its initial value of 2042 Pa within 80 seconds after the large strain (500%) was removed (Fig. 4(e)). In addition, the hydrogel exhibited strong self-healing performance, as evidenced by the 10 cut-healing cycles, in which it maintained over 80% of its initial ionic conductivity.

Expanding upon the ionic interaction approach in self-healing, Kim *et al.*^51^ discovered a self-healing ion conductor by integrating a copolymer of acrylic acid (AA) and 1-allyl-3-methylimidazolium bis(trifluoromethylsulfonyl)imide (AAHA) with a TFSI-based ionic liquid. The resulting ionoconductor leverages a unique ion-cluster-driven self-healing mechanism, where reversible electrostatic interactions between ionic groups enable rapid repair of mechanical damage at room temperature. Unlike conventional self-healing materials that require external stimuli, this system achieves approximately 90% mechanical recovery within one minute at 25 °C, while maintaining high stretchability (up to 1130%), conductivity and healing ability over six months without significant degradation due to moisture, oxidation, or mechanical stress. The material's robust mechanical and electrical performance enables its application in reconfigurable and wearable electronics, as demonstrated by stretchable electroluminescent devices that sustain repeated deformation and healing cycles.

##### 2.2.1.3. Ion-dipole or dipole–dipole interaction

Ion-dipole interaction involves an attractive force between a charged ion and a polar molecule.^52^ On the other hand, dipole–dipole association involves molecules that contain zwitterionic groups, in which a cationic and an anionic group coexist within the structure. The two oppositely charged ends induce a strong dipolar character (partial positive and negative) and thus allow them to interact with another polar molecule. To address the threats posed by the energy crisis, there has been a growing demand for fuel cells, which are seen as clean energy devices. Polymer electrolyte membrane (PEM) is the main component of fuel cells and should exhibit high ionic conductivity, durability, and low fuel permeability. A self-healing solid PEM, created by incorporating poly(vinylidene fluoride-*co*-hexafluoropropylene) (PVDF-HFP) into poly(ionic liquid) (PIL) matrices, was demonstrated by Gao *et al.*^53^ for flexible batteries. The interactions between imidazolium cations and dipoles in the polymer enhanced lithium ion transport, making the hydrogel more conductive. The ionic conductivity of the PEM was enhanced from 7.5 × 10^−5^ S cm^−1^ to 3.7 × 10^−4^ S cm^−1^ as the PIL content increased from 5 to 27 wt%. Most importantly, after 1 hour of scratch and recovery analysis at ambient temperature, the PEM showed no scratch (as shown in Fig. 4(f)), which could be explained by ion-dipole association, which enabled electrolyte self-healing and restored ionic conductivity.

Focusing on self-healing ionic conductors, Ming *et al.*^54^ leveraged ion-dipole interactions within a fluorinated poly(ionic liquid) copolymer to establish self-healing behaviour (Fig. 4(g) and (h)). The conducting polymer was cut into two pieces and then manually assembled them together. Upon self-healing at room temperature for 24 h, the conducting polymer regained ultrahigh stretchability, achieving 96% recovery in mechanical properties and intrinsic conductivity without additives. This characteristic simplified the material's composition and reduced potential issues related to evaporation or leakage, which are common in conventional conducting gels.

Textiles are a common choice for wearable electronic devices because of their lightweight, flexibility, and high surface area. However, textiles tend to fall off, leading to unstable sensitivity. Zhang *et al.*^55^ developed a sensitive ionic liquid building block comprising poly(vinylidene fluoride-*co*-hexafluoropropylene) (FE) and multi-cation ionic liquids with high-binding dynamic bonds. The authors reported that the ionic conductors showed enhanced mechanical properties without compromising ionic conductivity (3.4 × 10^−5^ S cm^−1^). Notably, the electronic conductors could endure multiple damage-healing cycles and readily self-heal under water through multivalent ion-dipole interactions, recovering 62% of their ionic conductivity. This feature is particularly beneficial for applications (such as biosensors and touch panels) in marine environments, where materials are often subjected to harsh conditions and must maintain functionality after damage.

It is unavoidable that wearable electronic devices (*e.g.*, communications systems, implantable bioelectronics, or prosthetics) are exposed to water, sweat, and high-humidity conditions. Therefore, Tolvanen *et al.*^56^ demonstrated a potential route using poly(3,4-ethylenedioxythiophene):poly(styrenesulfonate) (PEDOT:PSS) to confer self-healing under all of the aforementioned circumstances. PEDOT serves as an electronic conductor, increasing the electrode's capacity and rate capability, while PSS provides primary mechanical support and acts as a physical crosslinker. The material regained its original tensile and electrical characteristics in remarkably short times: less than 120 seconds for tensile properties and 900 seconds for electrical properties. This rapid recovery was attributed to the efficient self-healing dipole–dipole mechanism (O–H and O–B bonds) that operated autonomously without external intervention.

##### 2.2.1.4. Combination of ionic and hydrogen bonding

Despite the conflict between mechanical properties and healing efficiency, research on various self-healing mechanisms has become an opportunity to resolve this bottleneck. Some publications^57–60^ on self-healing are based on a combination of dipole interactions, imine bonds, and hydrogen bonding.

**Fig. 5:**
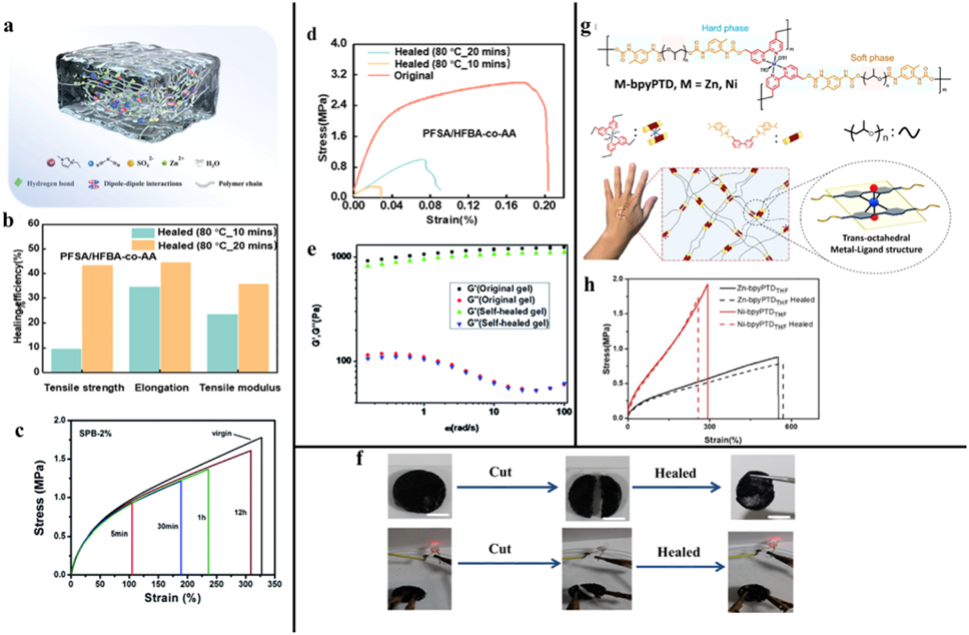
(a) Design of ionohydrogels. Reproduced with permission from *Small*,^57^ copyright 2021. (b) Self-healing efficiency of PFSA/HFBA-co-AA before and after healing in terms of mechanical properties, reproduced with permission from *ACS Omega*,^60^ copyright 2024. (c) Stress–strain curves of SPB-2% after different healing periods, reproduced with permission from *Journal of Materials Chemistry A*,^59^ copyright 2015. (d) Stress–strain curves of PFSA/HFBA-co-AA before and after healing, reproduced with permission from *ACS Omega*,^60^ copyright 2024. (e) Rheological results of the original and self-healed hydrogel, reproduced with permission from *RSC Advances*,^61^ copyright 2014. (f) Photographs demonstrating the self-healing behavior of PNIPAM/L/CNT hydrogel, reproduced with permission from *ACS Applied Materials & Interfaces*,^62^ copyright 2019. (g) Design concept and chemical structures of the self-healing and stretchable metal–ligand polymer, reproduced with permission from *Chemical Engineering Journal*,^64^ copyright 2022. (h) Stress–strain curves of the Zn-, Ni-bpyPTDTHF films before and after self-healing, reproduced with permission from *Chemical Engineering Journal*,^64^ copyright 2022.

Ionohydrogel, which differs from hydrogel by replacing water with ionic liquid and inorganic salt. It shows great potential in the electrochemical industry due to its excellent thermal and chemical stability, stretchability, and, most importantly, adjustable conductivity. Taking advantage of ionohydrogel, Huang *et al.*^57^ proposed a reliable and durable ionohydrogel from copolymerization of *N*-acryloyl glycinamide (NAGA) and 3-dimethyl(methacryloyloxyethyl) ammonium propane sulfonate (DMAPS) (Fig. 5(a)) as an electrolyte in zinc-ion batteries to power electronic devices. One of the standout features of the developed ionohydrogel is its remarkable ability to self-heal almost instantly by placing the cut surfaces in contact and allowing them to heal at room temperature for 10 min. Due to the presence of hybrid hydrogen bonds and dipole–dipole interactions between the pendant zwitterionic functional groups on the DMAPS component, the gel could stretch up to five times its original length, making it highly adaptable for various applications.

Wang *et al.*^58^ utilised oxidised nanocellulose and polyacrylamide, along with the organic polymer, polyaniline, as the conductive substance to form a self-healing conductive material. Surprisingly, the green material yielded fracture stress as high as 1.62 MPa (≈400%). In the meantime, the self-healing was achieved through hydrogen bonding and electrostatic interactions. The interactions provided the material with self-healing, restoring conductivity to more than 90% of its initial value after fractured pieces were reconnected at room temperature for 48 h. In addition, hydrogen bonding and electrostatic interactions enabled the self-healable polymer to achieve a maximum stress of 0.83 MPa.

Wang *et al.*^59^ created a rubber with special sensing capabilities that could heal itself *via* ionic hydrogen bonds formed by reactions between organic compounds. As shown in Fig. 5(c), they mixed amine- and carboxylic acid-functionalized polybutadiene (PB) to form a network that allowed the rubber to recover up to 90% of its original strength after 12 hours at room temperature. Despite its self-healing ability, the rubber had low strength, with a breaking stress of about 1.78 MPa, limiting its use in heavy engineering tasks.

In real-world fuel cell applications, PEMs undergo repeated swelling and deswelling, leading to the formation of microcracks and pinholes. Such degradations may cause fuel crossover, decrease fuel efficiency, and ultimately lower the power output. Mo *et al.*^60^ paved the way for the development of more durable and reliable PEMs by blending a self-healable copolymer consisting of hexafluorobutyl acrylate (HFBA) and acrylic acid (AA) into perfluorinated sulfonic acid (PFSA). The PFSA/HFBA-*co*-AA membrane encompassed a synergy of hydrogen bonds and ionic interactions, demonstrating remarkable recovery of mechanical strength, regaining 43.6% of its original tensile strength within just 20 minutes (Fig. 5(b) and (d)). This rapid recovery is a significant improvement over traditional PEMs, making them more reliable under operational stress.

##### 2.2.1.5. Hydrophobic association

Hydrophobic association is usually modulated by synthetic amphiphilic polymers that contain hydrophilic and hydrophobic segments. Amphiphilic polymers are usually created by incorporating hydrophobic monomers into the hydrophilic polymer network. The hydrophobic part tends to aggregate in water to avoid contact with it, which, in turn, acts as a crosslinking point for intermolecular non-covalent interactions to improve mechanical strength. These crosslinking points are also responsible for the self-healing process and can be re-mended.

Ferrocene (Fc) is used as a potential hydrophobic self-healing material in hydrogel due to its reversible redox behaviour. Li *et al.*^61^ prepared an anti-tumour agent by attaching Fc to chitosan (CS) *via* freeze-drying. Hydrophobic Fc groups aggregated and formed a crosslink to form a self-standing hydrogel. After the hydrogel was divided into two parts and reassembled to form a unity for 4 hours, the rheology analysis indicated that the storage modulus G′ of the repaired gel closely matched that of the original hydrogel (Fig. 5(e)). FcCS hydrogel exhibited quick recovery due to the reformation of reversible crosslinking points by hydrophobic Fc aggregates.

Deng *et al.*^62^ prepared a hydrogel for human motion sensing based on the hydrophobic association by conjugating nanoclay and multiwalled carbon nanotube (CNT) into *N*-isopropyl acrylamide (NIPAM) system. The hydrogel demonstrated a high value of elongation at break (1062%), indicating excellent flexibility and stretchability. The step-strain test was performed to investigate the healing ability. After 3 repeatable self-healing cycles, the storage modulus of the self-healing gel returned to nearly the same value as the original immediately. Also, the hydrogel was connected to a simple circuit to assess the self-healing application. The LED light was turned off when the hydrogel was divided, and the light was re-ignited at a similar intensity after the hydrogel self-healed (Fig. 5(f)).

##### 2.2.1.6. Metal-coordination bond

Metal-coordination bond is a robust non-covalent interaction in which a central metal atom or ion is surrounded by molecules or ions, known as ligands, that contain at least one donor atom with an available electron pair.^63^ The reversible coordination bonds between metal ions and ligands in the polymer matrix enable the material's self-healing. As the world moves into the era of the Internet of Things (IoT), merging electronics with biological skins has attracted tremendous attention. Pervasive efforts have been made to accommodate desirable properties, such as miniaturization, imperceptibility, transparency, and stretchability. In this regard, Han *et al.*^64^ provided insights on synthesizing a highly efficient self-healing film with exceptional mechanical strength from bipyridine (bpy)-capped polyurethane (PU) networks (Fig. 5(g)). The films were subjected to successive tensile tests after cyclic deformation, and because of the strong affinity of the metal–ligand coordination enabled by the metal ions (Zn^2+^ and Ni^2+^) and bpy ligands in the film, a self-healing efficiency of up to 93% was recorded within 12 hours (Fig. 5(h)). For instance, if a solar cell is transparent, it can be integrated into windows to generate energy without obstructing visibility. Such functionality is particularly valuable for applications in light-emitting diodes (LEDs) and photovoltaic devices.

For skin-inspired sensing, Chen *et al.*^65^ developed a novel approach by integrating aluminium ions and acetylacetonates (acac^–^) as dynamic metal salt crosslinkers into a system comprising phosphorus-rich small molecules (3N2AP) and pyridine-capped polyurethane-urea (PTD), which has been less explored compared to the use of transition or rare earth metal ions. The as-prepared polymer (Alac-3N2AP-PTD) was enriched by the behaviour of dynamic metal–ligand coordination (referring to Al^3+^ ions with acac^–^), which resulted in remarkable mechanical robustness (16.56 MJ m^−3^), as well as provided insight in impressive healing capabilities of 91.8% in term of mechanical strength. For validation, they also explored the direct application of the film for e-skin sensing on the human wrist by monitoring changes in relative resistance during wrist bending, and the results showed that the sensor's signals remained stable and reproducible.

While the stress sensor is vulnerable to mechanical damage, Li *et al.*^66^ modified methyl vinyl silicone rubber (MVQ) with 2-Isocyanatoethyl Acrylate (ICA) and dopamine (DOPA). The DOPA-ICA-MVQ elastomer was crosslinked *via* dynamic catechol-Fe^3+^ coordination bonds, endowing it with self-healing properties without compromising mechanical strength. The cuts on Fe(DOPA)_3_-ICA-MVQ composites were subjected to healing without external stimuli for 84 hours, during which they recovered 84% of strain and 91% of elongation. Notably, the authors integrated the self-healing sensor with carbon nanotube (CNT) film to investigate the electrical self-healing ability. With 20 layers of CNT, the resistance was as low as 10.2 kΩ, demonstrating a desirable, stable conductivity.

#### 2.2.2. Covalent bonding

##### 2.2.2.1. Diels–Alder (DA) reaction

Diels–Alder (DA) is a reaction between an electron-rich diene and an electron-deficient dienophile, forming a cyclohexene. DA reaction can be slow in the absence of a catalyst and under mild conditions. The most common DA pairings are maleimide and furan.

**Fig. 6:**
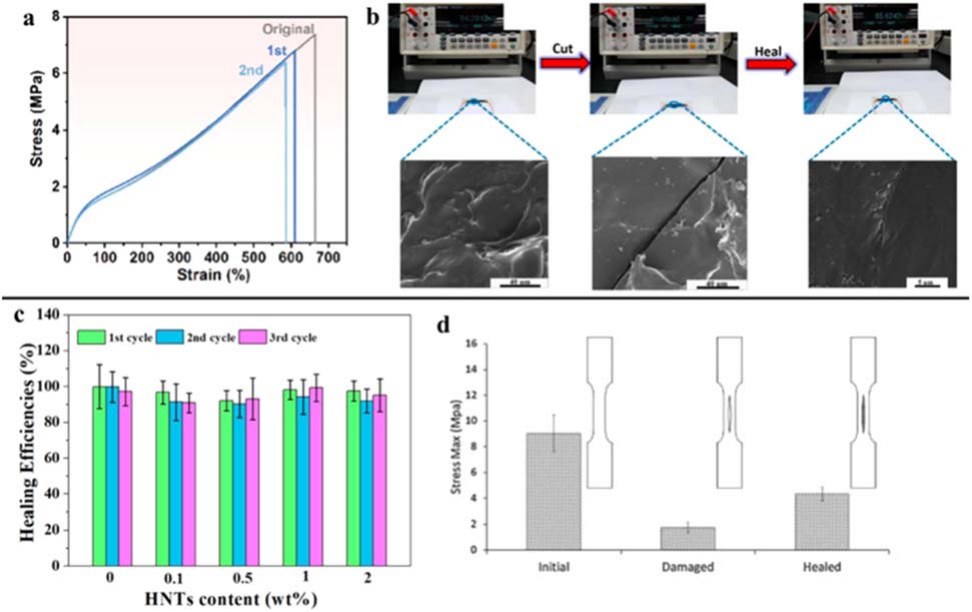
(a) The stress–strain curves of NRFB_3_ after self-healed, reproduced with permission from *ACS Applied Electronic Materials Journal*,^67^ copyright 2024. (b) Healing process of the flexible electronics based on DA bonds, reproduced with permission from *ACS Applied Materials & Interfaces*,^68^ copyright 2018. (c) Self-healing efficiency of polyurethane nanocomposites containing different amount of HNTs, reproduced with permission from *Polymer*,^69^ copyright 2020. (d) Maximum stress values of original, damaged, and healed polymers, reproduced with permission from *Progress in Organic Coatings*,^70^ copyright 2015.

Yan *et al.*^67^ designed a novel type of self-healing natural rubber (NRFB) that incorporated dual crosslinking using a monomer with double vinyl groups (HEF) and a Diels–Alder reaction between furan rings and maleimide. The NRFB exhibited impressive mechanical characteristics, achieving a tensile strength of 7.39 MPa and a breaking elongation of 664%. These properties make it suitable for applications that require durability and flexibility, which are critical for wearable technology. The authors found that NRFB exhibited fast and autonomous recovery in 20 minutes, showing self-healing efficiency of 91.9% and 88.5% in the first and second cycles of self-healing, respectively (Fig. 6(a)).

In an attempt to extend the applicability of graphene in flexible electronics, Li *et al.*^68^ modified a 3-dimensional graphene structure by filling the structure with furfurylamine (FA) and polyurethane to enhance crosslinking in the self-healing gel. The tests showed that gel with 2 wt% of graphene established better stretchability and conductivity. Based on the reversible Diels–Alder reaction, the cut-surface trace disappeared within 5 hours (Fig. 6(b)), demonstrating the gel's potential for applications in electronic skin and wound healing.

Lin *et al.*^69^ prepared a polymer composite *via* a facile solution casting method. Halloysite nanotubes (HNTs) were incorporated into a polyurethane with furan side groups to form a polymer composite, which was then further crosslinked with maleimide. The prepared polymer could respond to 2 cm-damage and realize self-healing. As shown in Fig. 6(c), the healed sample exhibited at least 90% self-healing efficiency in each healing cycle since DA bonds guide the polymers to amend themselves.

Postiglione *et al.*^70^ developed a polymer coating based on furfurylamine (FA) crosslinked with benzyl alcohol to improve self-healing efficiency. The experiment showed that the polymer could achieve rapid self-healing by closing cracks, achieving 48% self-healing efficiency at maximum stress (Fig. 6(d)). This could be explained by the mobility of the DA bonds across the interface, allowing multiple recoveries at the same scratched areas, demonstrating the effectiveness of the self-healing.

##### 2.2.2.2. Imine bond

**Fig. 7:**
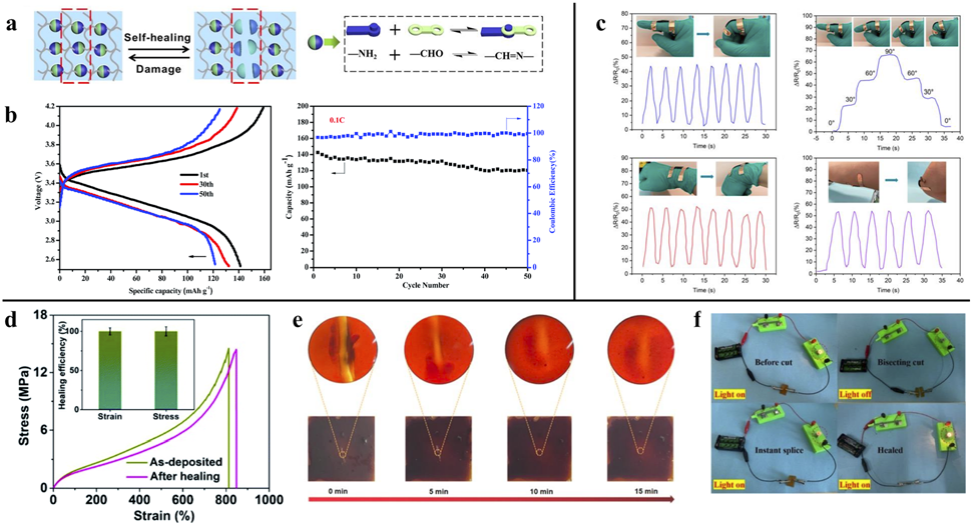
(a) Chemical structures of dynamic imine bonds and demonstration of healing process, reproduced with permission from *Nano Energy*,^71^ copyright 2021. (b) Charge/discharge performance and cycling performance of the battery, reproduced with permission from *RSC Advances*,^72^ copyright 2021. (c) Dynamic response of PDMS/CNT sensor for monitoring various human activities, reproduced with permission from *Molecules*,^73^ copyright 2023. (d) Stress–strain curves of PU before and after healing, reproduced with permission from *Journal of Materials Chemistry A*,^76^ copyright 2022. (e) Optical microscope images of PU film of the self-healing process, reproduced with permission from *Carbon*,^77^ copyright 2022. (f) Small bulb physical simulation experiment before and after healing. Reproduced with permission from *Journal of Polymer Science*,^79^ copyright 2024.

The imine bond is formed through the condensation reaction between an amine group (–NH_2_) and a carbonyl group (C=O), resulting in the formation of a C=N bond (imine group). In the context of self-healing conductors, imine bonds contribute to the healing process due to their dynamic covalent nature, which allows them to break and reform under certain conditions reversibly. This characteristic makes them ideal for self-healing materials, enabling damaged polymer matrices to recover and restore their mechanical and conductive properties. Yang and co-workers^71^ proposed a hybrid system consisting of a solar cell and a self-healable triboelectric nanogenerator (SH-TENG). TENG was constructed using self-healing polydimethylsiloxane elastomer (SH-PDMS), which served as a shielding layer for solar cells, safeguarding them from mechanical damage while maintaining their highly hydrophobic properties and ensuring continuous energy generation. Their findings revealed that SH-TENG fully self-healed within 30 minutes under ambient conditions after being scratched. This self-repair process was driven by the regeneration of imine bonds between active amino and aldehyde groups (Fig. 7(a)).

Nevertheless, over the past few decades, fragile solid electrolytes, which are inevitably bent and twisted during operation, have been a long-standing problem for lithium metal batteries. In the pursuit of high mechanical strength and self-healable solid polymer electrolyte, Cao *et al.*^72^ used a blend of glycol (PEG), diglycidyl ether of bisphenolA (DGEBA) and terephthalaldehyde (TPA), crosslinked with polyoxyethylenebis(amine) (NH_2_–PEG–NH_2_). This innovative polymer electrolyte exhibited ionic conductivity, up to 1.67 × 10^−4^ S cm^−1^, as the PEG content increased. It is worth mentioning that the reaction between amine and aldehyde groups forms an imine bond for self-healing. To validate the self-healing performance, the electrolyte was cut into two pieces and healed at ambient conditions for 30 minutes. The authors observed that the cut pieces fused into a single entity and achieved 86.7% self-healing efficiency in terms of discharge capacity (from 137.7 mAh g^−1^ to 119.34 mAh g^−1^) after 50 healing cycles (Fig. 7(b)).

Wang *et al.*^73^ incorporated dynamic imine bonds into polydimethylsiloxane (PDMS) networks by introducing carbon nanotube (CNT) and boroxine. The PDMS/CNT sample exhibited a tensile strength of 0.1082 MPa and an elongation of 349.50%. It was then attached to a finger to demonstrate its functionality as a strain sensor for detecting relative resistance changes and electrical signals associated with human joint motion. As depicted in Fig. 7(c), the recorded sensing signal remained stable when the finger was held at a fixed bending angle. Moreover, the PDMS/CNT elastomers exhibited excellent durability, allowing for multiple reprocessing cycles under mild conditions without significant loss of mechanical properties.

An interesting self-healable polydimethylsiloxane (PDMS) film with an extended aminopropyl group was designed by Yang *et al.*^74^ for application in human motion sensing. The film was constructed to self-heal at room temperature through reversibly imine bonds. The best results were obtained with a 0.8 molar ratio of IPDI to PDMS. Two cut films were autonomously reconnected in a water medium within 1 hour at room temperature, and the incision disappeared in 24 hours. The film also displayed up to 95% self-healing efficiency compared to the original film's tensile stress (400 kPa).

A new class of self-healing mechanisms that combines two reversible chemical interactions, imine bond, and hydrogen bonding, was introduced by Yan *et al.*^75^ for artificial skin. Terephthalaldehyde (TPA) and isophorone diisocyanate (IP) were added to poly(dimethylsiloxane) (PDMS), resulting in the formation of reversible imine and hydrogen bonds. The rubber achieved a maximum stretchability of 4000% without breaking, and the strength increased with increasing IP. The silicone rubber showed rapid self-healing (93% efficiency) within 3 hours at room temperature, whereas the plain PDMS film showed irreversible self-repair due to the absence of imine and hydrogen bonds.

##### 2.2.2.3. Disulfide bond

In dynamic covalent chemistry, disulfide bond has low bond energy and is prone to exchange reactions. It was then widely used in rubber (or polyurethane) to implant self-healing ability. When the rubber suffers damage, the rubber undergoes multiple times of healing by exchanging the disulfide groups in the crosslinked network. In the study by Kong *et al.*,^76^ they elucidated a novel type of stretchable self-healing conductors based on a dynamically crosslinked polyurethane (PU) polymer utilizing disulfide covalent adaptable networks (CANs). Designed for prostheses, soft robots, and health-monitoring applications, this highly self-healable PU exhibited an exceptionally low hysteresis of 3.8% and outstanding strain and stretchability of 812% (Fig. 7(d)).

Zheng *et al.*^77^ prepared perovskite solar cells from polyurethane (PU), and fullerene was grafted to the polymer *via* a disulfide bond. The resulting film exhibited high crystallinity and hydrophobicity, reducing the likelihood of breaking the chemical bonds in the original perovskite and thereby enhancing the stability of the solar cells. Nevertheless, the disulfide bond was broken during scribing, and, interestingly, after 15 minutes of the healing procedure, it guided the broken pieces to reconnect with the adjacent chain. As shown in Fig. 7(e), the PU film exhibited outstanding self-healing ability, completely repairing the surface defect.

Chou *et al.*^78^ designed a flexible, environmentally friendly, and durable TENG for energy harvesting from human motion. They fabricated TENG from zwitterionic polymer, [2-(methacryloyloxy) ethyl] dimethyl-(3-sulfopropyl) ammonium hydroxide (SBMA). The generator showed 250% elongation and managed to retain its original viscoelastic properties after three loading/unloading cycles, even after self-healing from bifurcating damage. The self-healing process involved double-terminal disulfide bonding, which migrated to the cleavage surface and healed the crash.

Another conductor based on disulfide interaction between 4, 4-diaminodiphenyl sulfide (DTDA), boronic acid (BA) and polyurethane (PU) was prepared by Chen *et al.*^79^ BA attachment enhanced the mechanical properties of PU from 18.76 MPa to 23.21 MPa due to the disulfide bond, which limits the slippage of interchain. The self-healing property was determined by comparing the tensile strength of the cut segments before and after 12 hours of self-healing. The spliced segments could bear 21.60 MPa before interface fracture, displaying a 93.06% self-healing efficiency. As shown in Fig. 7(f), the composite conductor was connected to a circuit before and after being healed. The conductor was responsive to damages, and it restored its electrical conductivity to light up the bulb.

Song *et al.*^80^ reported a simple way to obtain multi-responsive hydrogel by blending pluronic F127 and lipoic acid (LA). The dynamic disulfide interaction in the hydrogel could increase tensile strength from 0.025 MPa to 0.12 MPa as the LA mass fraction increased from 10 wt% to 20 wt%. Due to the dynamic network, the hydrogel achieved 81.2% self-healing efficiency of tensile strength within 20 minutes.

Intrinsic self-healing systems offer repeatable healing and are structurally integrated but may not match the rapid healing speed or electrical restoration efficiency, particularly in scenarios that demand immediate recovery under severe mechanical damage or large-area fractures, such as in high-performance power devices or critical circuit components. In such cases, extrinsic systems that use encapsulated healing agents can deliver a faster response by locally releasing pre-stored repair substances. Nevertheless, each system has a trade-off: intrinsic systems are better for flexible, repeatable self-healing, which is ideally suited for coatings and flexible electronics.

Table 1 summarizes the prominent materials used for various self-healing mechanisms.

**Table 1:** Summary of the prominent intrinsic self-healable materials in various applications

Materials	Self-healing mechanism	Healing efficiency (%)	Application/functions	References
CNT embedded poly(dimethylsiloxane)-isophorone bisurea-4,4′-methylenebis(phenyl urea) (PDMS-MPU_0.4_-IU_0.6_)	Hydrogen bond	100	Electronic conductor for electronic skin	81
Blending poly(vinylidene fluoride-*co*-hexafluoropropylene) (PVDF-HFP) into poly(ionic liquid) (PIL) matrices	Ion-dipole interaction	>97.1	Flexible batteries	53
Poly (vinylidene fluoride-*co*-hexafluoropropylene) (FE) and multi-cation ionic liquids	Ion-dipole interaction	61	Biosensors, touch panels and marine ships	55
µNi particles dopped complex	Hydrogen bond	90	Electronic conductor for pressure sensor	82
PU-DHP/Fe2	Hydrogen bond and metal-coordination	96	Electronic conductor for TENG	83
AgNWs embedded MDPB–FGEEDR copolymer	Diels–Alder reaction	97	Electronic conductor	84
AgNWs embedded PBPUU	Hydrogen bond and disulfide bond	∼77	Electronic conductor for electronic skin	85
CNT embedded PDMS-MPU_0.4_-IU_0.6_	Hydrogen bond	100	Electronic conductor for electronic skin	86
CNTs-PDA fillers doped PU inverse opal matrix	Hydrogen bond	90.9	Electronic conductor for human-motion sensor	87
PPBN-hydrogel	Hydrogen bond and metal-coordination	90.8	Conductor for strain and temperature sensor	88

## 3. Factors affecting self-healing efficiency

### 3.1. Healing period

A sample's recovery ability depends on molecular chain mobility. Complete healing requires sufficient mobility of the chains. Thus, the relationship between chain segment mobility and healing time was studied.

Wang *et al.*^73^ evaluated the healing efficiency of the silicon elastomer over different healing periods, ranging from 5 minutes to 2 hours. The cracks became narrower and shallower as healing progressed. The tensile properties can quantitatively determine the degree of healing. It is worth noting that the self-healing efficiency, calculated as the ratio of tensile strengths of the healed sample to that of the original, increased from 55.4% to 97.8% as the healing period extended from 5 minutes to 2 hours.

Peng *et al.*^89^ investigated the self-healing efficiency of ionic interactions and Diels–Alder in furan-functionalized methacrylate (FMA) networks under different healing durations. Based on the self-healing analysis, the bisected films partially and automatically recovered to their original shape within 17 hours. Notably, the healing behaviour improved as the healing period increased to 1 week, during which the scratch disappeared, indicating complete healing. Such healing ability could be ascribed to the reforming of both dynamic bonds, which required more time to diffuse across the fracture.

Patrick *et al.*^90^ reported that the healing performance could differ for horizontal and vertical channel designs. The healing agent (a polyurethane (PUR) foam formulation) delivered through horizontal channels exhibited rapid healing kinetics. The design captured over 75% recovery in both stiffness and fracture toughness within 1 h at room temperature.

Overall, these studies demonstrate that healing efficiency is strongly influenced by the duration of the healing process, with longer healing periods enabling more effective molecular diffusion and the reformation of dynamic bonds.

### 3.2. Concentration of healing materials

Reversible bonding allows the materials to break down and rejoin upon the attraction. Following this, the healing ability depends on the content of the functional material, which exhibits self-healing properties. Therefore, the healing ability can be tailored based on the characteristics of the healing agents within the matrix.

Zeng *et al.*^44^ adjusted the ratio between bis(2-hydroxyethyl) disulfide (HEDS) and terephthalaldehyde (TA) and found that self-healing efficiency could be increased to over 90% with an increasing number of imine bonds. The availability of the dynamic bonds allowed sufficient breaking tolerance so the broken chains could reform timely. However, as the hard domain (TA) content within the flexible polymer matrix (HEDS-based segments) increased, it hindered chain mobility, gradually weakening the material's relaxation behaviour.

Deng *et al.*^91^ focused on developing self-powered wearable electronics based on vitrimer with intrinsic self-healing properties. Due to the dynamic disulfide bonds within the vitrimer elastomer, the healing efficiency increased from 45% to nearly 100% as the pentaerythritol tetrakis(3-mercaptopropionate) content increased. The filler contributed to better recovery because the molecular chains could shuffle, leading to rearrangement.

He *et al.*^92^ studied the effect of the content of different fillers (polytetramethylene ether glycol (PEG) and hydrogenated 4,4′-methylene diphenyl diisocyanate) on the elastomer's self-healing efficiency. They found that increasing the PEG content increased disulfide bonds, thereby improving interactions between broken chains and allowing them to find their missing partners. Accordingly, the self-healing process became more orderly, and reversible properties could be achieved more quickly.

Table 2 implies that the self-healing period, materials, and mechanism involved could show conspicuous differences in self-healing efficiency. Both healing efficiency and healing period are strongly dependent on the type of reversible interactions employed. Materials that rely on hydrogen bonding or ionic interactions generally exhibit rapid healing with high recovery efficiency, whereas systems involving multiple bonding mechanisms or coordination interactions tend to require longer healing times.

**Table 2:** Comparison of the self-healing efficiency of different materials

Materials	Self-healing period	Self-healing mechanism	Self-healing efficiency (%)	References
Polyurethane-based polycaprolactone copolymer	22 s	Hydrogen bond	90	93
Polyacrylamide/κ-carrageenan	20 min	Hydrogen bond	>98	94
Histidine/brominated natural rubber	10 s	Ionic bond	93.98	95
Polymerization of zinc dimethacrylate in natural rubber	20 min	Ionic bond	100	96
Dihydroxybenzaldehyde (DHBA) modified PDMS/MWCNT	24 h	Hydrogen bonds, metal–ligand bonds and imine bonds	92.9	97
PVDF-HFP, together with [EMI]^+^ [TFSI]^−^	24 h	Ion–dipole interactions	99.1	98
PAA-LMs/rGO	24 h	Ionic and metal-coordination interactions	92	99
Lipoic acid	14 h	Disulfide bonds, hydrogen bonds, and metal-coordination bonds	86	100

## 4. Application

### 4.1. Signal transmission

There is a significant emphasis on incorporating self-healing material into human movement and postural evaluator to prevent performance failures such as malfunction or degradation of signal transmission, which result from repeated stretching, bending or impact during body motion. Such failures compromise the accuracy, reliability, and continuous operation. Therefore, self-healing conductors help maintain system integrity, ensure consistent signal monitoring, and understand the movement characteristics accurately.

In terms of energy harvesting devices, Zhang *et al.*^101^ introduced self-healing ionic hydrogel, which was composed of polyacrylamide (PAM), tannic acid (TA), sodium alginate (SA) and MXene (PTSM hydrogel), in flexible TENG. The PTSM hydrogel extended TENG's stretching capacity (from 5 mm to 200 mm) and endowed outstanding flexibility. Based on the self-healing testing, PTSM hydrogel could recover up to 91.5% of tensile properties after 4 hours of healing at room temperature. They also fixed the self-healing TENG in front of the glove-based Human Machine Interface (HMI) and analyzed hand movement information. The results showed that the intelligent glove achieved 98.7% accuracy by observing the output voltages for different finger gestures.

Wang *et al.*^102^ prepared a self-healing TENG using polyurethane/cellulose elastomers (PUBES) for target shooting in military training. PUBES were placed at −10 °C for 6 hours to observe the functionality of the TENG at low temperatures, in which the PUBES demonstrated good flexibility of 1100%. The PUBES also showed consistent voltage output at different impact frequencies. The analysis indicated that the TENG could reduce detection errors and transmit electrical signals accurately at low temperatures. PUBES also exhibited 93% self-healing efficiency through dynamic hydrogen and disulfide bonds at low temperatures.

### 4.2. Healthcare

Electronic skin (E-skin) mimics human skin's functions, which serve as an essential barrier to interaction with the environment. It was equipped with various sensory receptors to detect external stimuli (*e.g.*, touch, pressure, and temperature), just as natural skin does. When creating E-skin, the materials must be conductive, durable enough to withstand wear and tear, have reliable sensitivity, and be stretchable for real-world applications. Conductive hydrogels are promising materials due to their inherent stretchability and transparency. However, the flexible and wearable hydrogel tends to suffer damage; thus, its self-healing property could extend its lifespan.

Zheng *et al.*^103^ designed a wearable and stretchable E-skin based on a glycidyl methacrylate (GMA)-modified silk fibroin hydrogel with self-adhesive properties. In their research, they found that the E-skin could withstand pressures ranging from 0.1 to 10 kPa, demonstrating excellent environmental stability. To support the e-skin's ability to respond to gesture recognition, the e-skin was subjected to sensing, and the signals were maintained, indicating that the e-skin was sensitive to detecting different gestures. The e-skin also demonstrated good durability, enabling reconnection of the fracture parts without external stimulus at ambient conditions.

Han *et al.*^104^ proposed a self-healing gelatin/polyacrylicacid (PAA) for e-skin. The PAA system achieved rapid self-healing within 2.5 minutes at ambient temperature, achieving 800% elongation. The e-skin demonstrated strong sensing capabilities, increasing voltage with increasing touch pressure. This makes it useful for devices that need to interact with humans, such as wearable technology (*e.g.*, skin sensors).

Mondal *et al.*^105^ demonstrated autonomous self-healing in non-centrosymmetric polar single crystals of 1-(9-anthryl)ethanol (AEL), a simple and readily accessible organic molecule, for applications in wearable health-monitoring devices such as respiration tracking. Under external mechanical stress below 10 mN, fractured crystal fragments healed autonomously within 20–100 ms *via* self-actuation motion. The self-healing behavior was confirmed by single-crystal X-ray diffraction (SCXRD) and confocal microscopy, using the crystals' intrinsic fluorescence. Healing was also observed in crystals adhered to a flexible substrate subjected to qualitative bending for 3 repeated cycles.

### 4.3. Wireless communication system

An antenna enables electronic devices to communicate wirelessly by converting electrical signals into electromagnetic waves, such as radio and microwave signals. In contrast, communication systems that use visible or infrared light, including fiber optics and laser-based technologies, rely on specialized optical devices rather than conventional antennas. With that said, the modulated signal is transmitted from a transmission antenna to a receiving antenna, where it is demodulated and decoded.

An *et al.*^106^ encapsulated self-healing fluoroelastomer (SHFE) containing poly(vinylidene fluoride-*co*-hexafluoropropylene) (PVDF-HFP), which repaired damage to the antenna *via* dipole–dipole interactions. For the self-healing analysis, the SHFE was cut in half and reconnected. The reattached film exhibited excellent stretchability of 450% or more. SHFE was fixed in the RFID reader to measure the air-gap recognition. Based on the measurement results, the antennas could detect magnetic resonance from a greater distance (90 mm) than the commercial one (70 mm).

Impressively, Yu *et al.*^107^ designed a smart, self-healable plasmonic metamaterial by embedding an epoxy-polyimide composite into microwave transmission lines. The film was cut in half to visualize its self-healing ability, and the microscopic image was observed when the broken parts were brought into contact. The self-healed film remained intact after stretching. The self-healing could be explained by reversible imine bonds formed, as the polyimide promotes reliable adhesion between substrates and the hydrogel across different temperatures. Based on the oscillation frequency of the continuous step strain measurement, the self-healed film demonstrated stable microwave transmission, indicating the potential application of the epoxy-polyimide in wireless communication systems.

## 5. Challenges, opportunities, and sustainability

In terms of extrinsic self-healing, challenges related to healing agent depletion and the repeated replenishment of multiple healing agents in existing architectures remain significant. One promising strategy to address these limitations is the adoption of two-part healing systems, which may potentially provide unlimited self-healing capability.^63^ Such advanced configurations would allow greater flexibility in selecting healing reactions tailored to specific applications. However, incorporating large amounts of external healing agents into the matrix to avoid insufficient healing can introduce additional challenges, as excessive loading may compromise the mechanical properties and conductivity of the composites.^108^ Therefore, the concentration, distribution, and chemistry of the self-healing agents must be carefully optimized according to the functional requirements of the targeted electronic application.

Another critical challenge lies in the long-term stability of autonomously self-healing polymer matrices under diverse environmental conditions. Many systems rely on dynamic interactions, such as hydrogen bonding, which are inherently sensitive to moisture, temperature fluctuations, and repeated swelling–deswelling cycles.^109^ These effects may lead to surface aging, mechanical degradation, or reduced healing efficiency over time.^110,111^ In addition, healing speed and recovery efficiency often deteriorate after multiple healing cycles due to irreversible structural changes or the gradual exhaustion of active healing sites.^112^

Despite these challenges, self-healing materials offer substantial opportunities to advance sustainable electronic devices. Future opportunities in self-healing electronics lie in developing environmentally robust and sustainable material systems. These include moisture-insensitive dynamic bonds, covalent adaptable networks, and bio-based or recyclable healing agents that maintain healing performance over extended operational lifetimes.^113,114^ By extending device lifespans, reducing material waste, and minimizing maintenance and replacement frequency, self-healing electronics can directly contribute to resource efficiency and environmental sustainability.

## 6. Conclusions

Many scholars have noted that self-healing properties are crucial for developing electronic products, as they are inherently vulnerable to damage. This review assessed various autogenous self-healing methods and mechanisms in conductive electronic products. Intrinsic self-healing materials can heal themselves repeatedly without needing healing agents, where the healing process depends on the inherent component or originates from the incorporated filler. Intrinsic self-healing can be adapted in the form of non-covalent and covalent bonding, in which non-covalent bondings are weak interactions and render stronger toughness after repairing. On the other hand, extrinsic self-healing is limited by the availability of the incorporated healing agent stored in the capsule or vessel: once the damage is healed, the capsule or vessel is out of service. The review found that the self-healing performance is described based on the functionalities of the material, such as viscoelasticity, conductivity, mechanical measurement (tensile strength or elongation), and the ability to recover the original properties after damage. The measurements are not fully standardized, so comparing the self-healing efficiency may be difficult, leading to variations in experimental results. Future research directions in self-healing electronic materials should provide insights regarding challenges such as long-term stability, healing efficiency under extreme conditions, and scalability for commercial applications.

## Author contributions

Wei Wuen Ng: data curation, formal analysis, investigation, methodology, validation, visualization, writing – original draft. Wei-Hsin Chen: conceptualization, formal analysis, funding acquisition, investigation, project administration, resources, software, supervision, writing – review & editing. Hui San Thiam: formal analysis, investigation, writing – review & editing. Steven Lim: formal analysis, investigation, writing – review & editing. Yi-Kai Chih: formal analysis, investigation, writing – review & editing. Yean Ling Pang: formal analysis, investigation, writing – review & editing.

## Conflicts of interest

There are no conflicts to declare.

## Data Availability

No primary research results, software or code have been included, and no new data were generated or analysed as part of this review.
